# Laparoscopic Pielolitotomy: An option for the management of pelvic kidney stones

**DOI:** 10.1590/S1677-5538.IBJU.2019.0148

**Published:** 2020-02-20

**Authors:** Artur de Oliveira Paludo, Antonio Rebello Horta Gorgen, Marcio Araldi, Patric Machado Tavares, Nelson Sivonei Batezini, Tiago Elias Rosito

**Affiliations:** 1 Hospital de Clínicas de Porto Alegre Porto AlegreRS Brasil Hospital de Clínicas de Porto Alegre, Porto Alegre, RS, Brasil;; 2 Universidade Federal do Rio Grande do Sul – UFRS Porto AlegreRS Brasil Universidade Federal do Rio Grande do Sul – UFRS, Porto Alegre, RS, Brasil

## Abstract

**Introduction:**

Minimally invasive treatments such as extracorporeal shock wave lithotripsy and percutaneous nephrolithotripsy are standard procedures for the management of renal stones (1). However, renal position and rotation defects may significantly interfere in the results of these treatments (2). Open surgery has always been an option for these cases, but with the advancement of laparoscopy in the last decades, laparoscopic pielolitotomy has become a good alternative for approaching kidney stones in abnormal renal rotation and position (3).

**Materials and methods:**

A 42-year-old male patient with a 2.2cm stone in the left pelvic kidney was submitted to laparoscopic pielolitotomy after extracorporeal schok wave lithotripsy failure and difficulty in access for percutaneous nephrolithotripsy. We did not have access to flexible ureteroscopy for this case.

**Results:**

The surgical time was 150 minutes. An antegrade JJ stent was inserted and renal pelvic suture was performed with vicryl 4-0. There was no need for opioids and patient was discharged on the first postoperative day. The JJ stent was removed after 1 month, with complete resolution of the clinical symptoms.

**Conclusions:**

Laparoscopic pielolitotomy is an excellent treatment alternative for patients with large stones in pelvic kidney.
